# Monitoring and evaluation of disaster response efforts undertaken by local health departments: a rapid realist review

**DOI:** 10.1186/s12913-017-2396-8

**Published:** 2017-06-29

**Authors:** Kate Gossip, Hebe Gouda, Yong Yi Lee, Sonja Firth, Raoul Bermejo, Willibald Zeck, Eliana Jimenez Soto

**Affiliations:** 1 School of Public Health, The University of Queensland, Level 2, Public Health Building (887) Corner of Herston Road and Wyndham Street, Herston, QLD 4006 Australia; 2 Policy and Epidemiology Group, Queensland Centre for Mental Health Research, The Park Centre for Mental Health, Level 3, Dawson House, Wacol, QLD 4076 Australia; 3 Global Burden of Disease Group Melbourne School of Population and Global Health, The University of Melbourne, Level 5, Building 379, 207 Bouverie Street, Carlton, VIC 3010 Australia; 4 UNICEF Philippines Country Office, 31st Floor, Yuchengco Tower Rizal Commercial Banking Corporation (RCBC) Plaza 6819 Ayala Avenue corner Gil Puyat Avenue Makati City, 1200 Makati, Philippines; 5 Institute of Tropical Medicine Antwerp, Nationalestraat 155, 2000 Antwerp, Belgium; 6 Department of Obstetrics and Gynecology, Medical University of Graz, Auenbruggerplatz 14, A-8036 Graz, Austria; 7 Abt Associates Australia, 5 Gardner Cl, Milton QLD, Brisbane, 4064 Australia

**Keywords:** Monitoring and evaluation, Disaster response, Health departments, Lessons learned, Rapid realist review

## Abstract

**Background:**

Local health departments are often at the forefront of a disaster response, attending to the immediate trauma inflicted by the disaster and also the long term health consequences. As the frequency and severity of disasters are projected to rise, monitoring and evaluation (M&E) efforts are critical to help local health departments consolidate past experiences and improve future response efforts. Local health departments often conduct M&E work post disaster, however, many of these efforts fail to improve response procedures.

**Methods:**

We undertook a rapid realist review (RRR) to examine why M&E efforts undertaken by local health departments do not always result in improved disaster response efforts. We aimed to complement existing frameworks by focusing on the most basic and pragmatic steps of a M&E cycle targeted towards continuous system improvements. For these purposes, we developed a theoretical framework that draws on the quality improvement literature to ‘frame’ the steps in the M&E cycle. This framework encompassed a M&E cycle involving three stages (i.e., document and assess, disseminate and implement) that must be sequentially completed to learn from past experiences and improve future disaster response efforts. We used this framework to guide our examination of the literature and to identify any context-mechanism-outcome (CMO) configurations which describe how M&E may be constrained or enabled at each stage of the M&E cycle.

**Results:**

This RRR found a number of explanatory CMO configurations that provide valuable insights into some of the considerations that should be made when using M&E to improve future disaster response efforts. Firstly, to support the accurate documentation and assessment of a disaster response, local health departments should consider how they can: establish a culture of learning within health departments; use embedded training methods; or facilitate external partnerships. Secondly, to enhance the widespread dissemination of lessons learned and facilitate inter-agency learning, evaluation reports should use standardised formats and terminology. Lastly, to increase commitment to improvement processes, local health department leaders should possess positive leadership attributes and encourage shared decision making.

**Conclusion:**

This study is among the first to conduct a synthesis of the CMO configurations which facilitate or hinder M&E efforts aimed at improving future disaster responses. It makes a significant contribution to the disaster literature and provides an evidence base that can be used to provide pragmatic guidance for improving M&E efforts of local health departments.

**Trial registration:**

PROSPERO 2015:CRD42015023526.

## Background

The Sendai Framework for disaster risk reduction 2015–2030 notes that natural disasters have caused more than $US 1.3 trillion worth of damage, affected 1.5 billion people and led to approximately 700,000 deaths over the past decade [1]. Poor, vulnerable and marginalised populations in low and middle income countries (LMICs) of East Asia and the Pacific are among those disproportionately affected by natural disasters [2]. Local health departments are often at the forefront of an emergency response, attending to both the immediate trauma and long term health consequences resulting from a disaster. Evaluating a department’s response to a disaster can offer insights into what supports or hinders the successful delivery of health services during a disaster [3].

From an evaluation perspective, substantive work has been undertaken recently to unpack the theory of change behind disaster efforts in general. The most prominent examples are the disaster logic model and the five frameworks developed by Birnbaum and colleagues [4–8]. They provide a systematic approach to assess the how and why disaster interventions lead to specific outcomes. However, very little is known about whether the M&E of disaster response efforts leads to better outcomes in terms of improved practice.

Monitoring and Evaluation (M&E) can be defined as a quality improvement process which health departments use to monitor, measure and assess performance. Evaluation reports are intended to identify strengths and weaknesses of a particular response and enable practical lessons to be learned and applied [9, 10]. These lessons feed into future strategic planning and are critical to both: formulating and revising policy; and refining improvement strategies [10, 11]. That being said, existing evidence demonstrates that lessons are not always learned from past disasters [4, 12–15]. If this continues to be the case, the priorities for action established by the Sendai Framework will fail to materialise. By not learning from past mistakes, key stakeholders will be unable to: improve their understanding of local disaster risks; strengthen local governance; improve the return on their increased investments; and enhance future disaster response efforts.

Savoia and colleagues [14] found that the same failure to learn lessons from previous disasters occurred during the public health emergency responses to three separate hurricanes in North America (i.e., Katrina 2005, Gustav 2008 and Ike 2008). Despite documentation of the problems, public health response procedures were not revised resulting in the same challenges being experienced years later during the emergency response to the H1N1 pandemic in 2009–2010.

This demonstrates that the failure to act on problems identified from past experiences can leave health systems and communities susceptible to the recurrence of the same problems during future events [4, 12–15]. A more recent example of this can be found in the response to Super Typhoon Haiyan, one of the most destructive typhoons recorded, which made landfall in the Philippines on November 2013. Following Haiyan, a stocktaking exercise was undertaken by the Philippine Department of Health and UNICEF to review the health response in the most affected regions. This exercise revealed that a number of provinces shared common challenges in responding to Haiyan, several of which had been experienced in previous disasters. Although the Philippine Department of Health conducted post-incident evaluations after each previous disaster, it was acknowledged that these efforts did not necessarily lead to improved disaster response procedures in the present circumstances (personal communication).

The aim of this study was to gain an understanding of why M&E efforts undertaken by local health departments often fail to improve future disaster responses. We used a rapid realist review (RRR) approach which adopts a realist perspective to knowledge synthesis to identify context-mechanism-outcome (CMO) configurations. The CMO configurations identified in the literature provide insights into why and how M&E is likely to work [16]. These findings can be used to help guide local health departments to improve M&E processes.

## Methods

### Overview

Our methods followed the six stages of realist evaluation outlined by Pawson [17] [1]: identify the research question [2]; formulate a theoretical framework [3]; search for primary studies [4]; select and appraise the quality of studies [5]; extract, analyse and synthesise the relevant data; and [6] refine the theoretical framework. Our research was also guided by the Realist and Meta-narrative Evidence Synthesis: Evolving Standards (RAMESES) publication standards [18].

### Identification of the research question

This study was conducted in response to the findings of the stocktaking exercise conducted after super typhoon Haiyan, which highlighted that previous post-disaster evaluations had not led to improvements in existing disaster response procedures. We consulted with a local reference group in the Philippines to clarify the focus of the review and determine what knowledge would be most useful to their efforts to improve future disaster responses. Our primary research question asked: how, why and under what circumstances can local health departments use M&E interventions to improve future disaster responses based on past experiences? We aimed to explore these questions by [1]: examining the contextual factors operating within M&E; and [2] identifying and analysing the mechanisms which facilitate or hinder successful M&E processes.

### Formulation of the theoretical framework

**Fig. 1:**
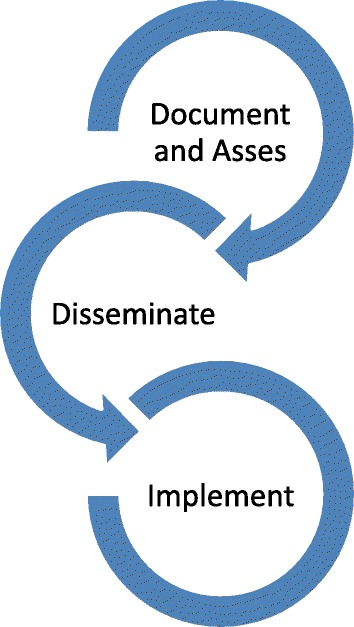
Monitoring and Evaluation framework

The theoretical framework for this study was developed based on a broad search of peer reviewed and grey literature describing health departments’ experiences with M&E. We formulated a framework based on the three fundamental stages of an M&E cycle that should be sequentially completed in order to successfully learn from past experiences – i.e., document and assess, disseminate and implement (Fig. 1). We used this framework to guide our examination of the literature and to analyse any constraining or enabling features which influence outcomes at each stage of the M&E cycle.

Realist evaluation is a theory driven approach which uses context-mechanism-outcome (CMO) configurations to understand the causal patterns underlying the success or failure of an intervention. CMO configurations explain how particular contexts (c) generate casual mechanisms (m) that produce an outcome (o) within an intervention [19]. A brief description of how disaster responses can be understood with respect to each stage within the M&E cycle is outlined below.

Stage 1 – Document and assess. The first stage of the M&E cycle is to document and assess the response to the disaster. This involves using a systematic approach to record information during and immediately after the response effort. This information is then reflected upon by personnel who are trained to conduct an assessment of the response. The goal of the assessment is to identify the fundamental strengths and weaknesses of a recent response to a natural disaster and propose recommendations to optimise systems performance [20]. These insights provide an evidence-base for the development of policies that support the improvement of future disaster response processes [21].

Stage 2 – Disseminate. Due to the infrequent nature of major natural disasters it is important that the lessons learned from one disaster response are disseminated effectively. However, the literature indicates that lessons are rarely communicated to their intended audience and that processes to promote the dissemination and uptake of lessons are weak [18]. Widespread dissemination of reports would ensure that the lessons learned during one disaster could be shared and used to improve disaster response practices for future events.

Stage 3 – Implement. The final stage of the M&E cycle is to use the lessons identified in the report to design and implement changes to improve disaster response protocols. While there is much attention given to the need to continually improve response efforts, research indicates that minimal resources are invested into creating the capacity for improvement [22]. Consequently, it has been found that many health departments have failed to act on lessons identified during previous disasters [23].

### Search for primary studies

**Fig. 2:**
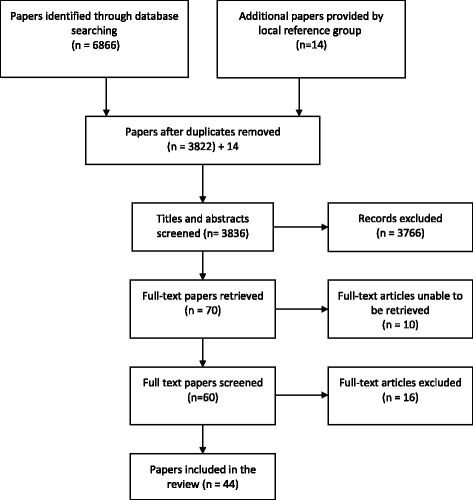
Search Strategy

The initial systematic search of the literature focussed on retrieving papers that investigated how health departments use M&E to improve disaster response efforts. A search strategy was developed which focused on three main concepts; health departments; M&E; and disaster response (Table 1 and Fig. 2). During the preliminary stages of developing the search strategy, we found very few papers examining how local health departments reflect on and analyse their response to a disaster. However, there appeared to be a significant degree of research on public health emergency preparedness. We postulated that many of the successes and challenges that local health departments experience when monitoring and evaluating the quality of emergency preparedness would be similar to the monitoring and evaluation of response efforts. Therefore, we included the term “preparedness” in our search strategy to enhance the body of literature to be analysed.

**Table 1:** Search terms used

Disaster
AND quality OR monitoring OR evaluation OR “after action report” OR “after-action-report” OR “after action review” OR “after-action-review” OR “after event report” OR “after-event-report” OR “after event review” OR “after-event-review” OR drills
AND response OR recovery OR preparedness OR management
AND Health

The same search strategy was used to explore electronic databases (i.e., *PubMed, Embase, Scopus* and *Web of Science*), in addition to Google and Google Scholar to retrieve any grey literature. We restricted our search from the year 2000 to the present as our preliminary scoping indicated that the majority of the literature describing the role of health departments in disasters emerged after the terrorist attacks on the United States in September 2001. There were no restrictions applied to the type of publication, however we only included papers written in English. The database searches concluded in May 2015 once a saturation point was reached (i.e., when the literature no longer provided new knowledge or added to our understanding) [19].

Consistent with realist evaluation our research involved collaboration with a local reference group who provided input throughout the review process. This local reference group included partners from the Philippines with expertise in disaster management. These partners facilitated the efficient identification of pertinent documents that would aid our research including disaster management policies and guidelines currently in use by health departments in the Philippines.

### Selection and quality appraisal of studies

An inclusion criteria was established after discussions with the local reference group about what would be most useful in terms of M&E for local health departments in the Philippines (Table 2). All titles and abstracts were independently assessed by two reviewers. Full text papers were retrieved and initially examined by one author to ensure that inclusion criteria were met. A second author then cross checked the selected papers before they were approved for review.

**Table 2:** Inclusion criteria

Inclusion	Exclusion
Papers which examined at least one stage of the theoretical framework (document and assess; disseminate; or implement)	Papers which focused on instruments used for the evaluation or adoption of performance standards, which are highly specific to the hospital setting
Papers which examined the evaluation of quality improvement efforts at the hospital level were included if their content was deemed to be applicable to health departments in general	Papers which focused on evaluating elements of preparedness (i.e. facilities or training of health personnel) or were reporting the health outcomes after a disaster rather than the response itself
	Papers which focused on research methods and metrics for the evaluation of disaster management and did not explore programme or policy aspects of the evaluation
	Papers which reported the results of an evaluation but did not provide a description of how the evaluation was conducted, or how the lessons learned were disseminated or incorporated into future plans

In realist evaluation, quality appraisal takes place during the synthesis stage and is guided by the concepts of *relevance* and *rigour* to determine whether potential information is fit for purpose [18, 19, 24]. When sifting through extracted information we first considered whether the information was relevant – i.e., does this information aid our understanding of potential mechanisms? Secondly we made judgments on whether the information provided sufficiently detailed descriptions of potential mechanisms – i.e., does this information contain rich detail that points to the mechanisms which link context and outcome?

The most relevant and detailed evidence featured more heavily in our analysis than papers containing weaker contributions. Realist evaluations do not typically apply a formal tool or checklist to assess potential biases. We did, however, consider elements of methodological soundness to aid in our quality appraisal (e.g. research question, study design, sample description, data collection procedure and data analytic technique). We included information contained within opinion pieces if such information was supported by or consistent with the findings of other empirical papers.

### Extraction, analysis and synthesis of relevant data

One reviewer read each paper and recorded the following details in an extraction template: country setting; level of health system under investigation; type of disaster; stage of the theoretical framework being evaluated (document & assess, disseminate and implement); and the barriers and facilitators of M&E. During this stage, we identified recurring contextual themes across papers. To further investigate these themes, each paper was re-read and important information regarding each contextual theme was transposed into another data extraction template. In this second iteration of data extraction we aimed to identify any prominent recurring patterns of contexts and outcomes, known as demi-regularities [18].

For example, we found that when health departments assure personnel that they will not be penalised for mistakes (C) the documentation and assessment of a disaster response is likely to be more accurate and thorough (O). We synthesised the extracted information to generate CMO configurations which explain causal pathways whereby certain contexts influence mechanisms that trigger positive or negative outcomes at each stage of M&E. In this particular example, our synthesis revealed that when health departments assure personnel they will not be penalised for mistakes (C) an organisational culture of learning is likely to be established which encourages staff motivation and willingness to engage with M&E (M). This would, in turn, result in more accurate and thorough documentation and assessment of the disaster response (O). We repeated this process of investigation and deduction for each of the demi-regularities found within the literature. Emerging findings were regularly shared and discussed with the review team to ensure the validity of inferences made.

### Refinement of the theoretical framework

In realist reviews the synthesis of information is used to refine the theoretical framework to determine what works for whom, how and under what circumstances [19]. We used the evidence synthesised in the review to fine tune our understanding of how M&E can be successfully used to improve future disaster response efforts.

## Results & findings

### Description of primary studies included in the analysis

Of the 44 papers included in this review, 23 made a significant contribution to our understanding of CMO configurations. Our quality assessment found that while some papers contained important contextual information regarding the current state of M&E used in disasters, they did not include any relevant information that could be analysed against our theoretical framework. These papers were deemed to possess insufficient detail to be included in the evidence synthesis. However, these papers were not excluded from the full review as the underlying contextual information contained in these studies aided our understanding of the current context of M&E approaches that have been used by health departments.

The following sections describe the CMO configurations identified within the demi-regularities in each stage of the M&E cycle (a summary of the CMO configurations is presented in Table 3).

**Table 3:** Context-Mechanism-Outcome configurations

Stage of M&E	Context	Mechanism	Outcome
Document and assess	Assurance that staff will not be penalised for mistakes.	A culture of learning is instilled in the health department	More accurate and high quality documentation and assessment of disaster response
	Training in the procedures and skills for documenting and assessing a disaster response are embedded into regular work	Provides staff with an opportunity to practice and become proficient in the analytical skills required to successfully document and assess their response.	Better prepared staff
	Using external partners to document and assess the disaster response	External partners have the time, knowledge and skills to thoroughly document and assess a response	Improved depth and quality of documentation and assessment
Disseminate	Disseminating standardised reports	Allows all health departments to perceive the experiences of others as relevant to their own work	Promotes inter-agency learning
Implement	Positive leadership attributes	Establishes a culture of quality improvement within the health department which increases commitment	Promotes successful implementation of changes to improve performance
	Shared decision making structures	Fosters a sense of ownership and responsibility amongst personnel which increases motivation and commitment	Promotes successful implementation of changes to improve performance

### Demi-regularities identified in the ‘document and assess’ stage of the monitoring and evaluation cycle

There were three demi-regularities identified within the document and assess stage of the M&E cycle.

The first demi-regularity in the document and assess stage was, ‘When health personnel are assured that there will be no punitive action for reporting mistakes they are more likely to accurately document the disaster response’. A number of seminal papers identified through this review highlighted that health personnel are reluctant to formally acknowledge and document errors made during a disaster response due to potential ramifications (e.g., liability, concerns about future funding and career advancement) [13, 25, 26]. However, concealing such errors leads to an inaccurate representation of the response and hinders improvement efforts. Seid and colleagues [22] found that an organisational culture which fosters openness, collaboration, teamwork, and learning from mistakes to be optimal for quality improvement.

When evaluation reports are driven by quality improvement purposes, health personnel are more likely to identify the potential pitfalls which may adversely affect a response to a future event [14]. We infer that within the context of a supportive and open workplace a culture of learning is instilled within the health department. This mechanism is likely to result in more accurate documentation and assessment of the disaster response as health personnel feel comfortable to report errors.

The second demi-regularity in the document and assess stage was, ‘Embedded training leads to more accurate documentation and assessment of disaster responses’. Accurately documenting and assessing a response to a disaster involves identifying strengths and weaknesses in the response and devising corrective actions which may improve future performance [13]. Due to the infrequency of large-scale disasters, health personnel have limited opportunities to learn and practice the analytical skills required to accurately document a response. This limitation may explain the lack of in-depth, quality insights contained in evaluation reports that have been acknowledged in the literature [13, 27]. Several of the key papers identified in this review noted that embedded training has proven to be a useful method for familiarising staff with the procedures that should be enacted when responding to a disaster situation [3, 22, 23, 28].

For example, in Nelson et al. [23], one of the most informative papers of this review, it was recounted that some public health departments across the United States have used annual influenza clinics to practice mass dispensing procedures, which may be required in a disaster situation [23]. Similarly, local health departments could use embedded training to provide personnel with opportunities to practice documenting the delivery of routine health services and considering how procedures may be improved. This process would train personnel to be proficient in the analytical skills required to successfully document and assess a disaster response. This mechanism is likely to result in more accurate and thorough documentation and assessment of future disaster response efforts.

The third demi-regularity in the document and assess stage was, ‘Partnering with external organisations improves the depth and quality of documentation and assessments’. In the aftermath of a disaster, health personnel are under extreme pressure to deliver emergency health services, minimise disruptions to regular health services and generate evaluation reports [29, 30]. The literature indicates that assessments conducted immediately after a disaster or after the termination of an emergency drill, are more likely to capture accurate narrative information that may otherwise be lost over time [9, 20, 31]. Some health departments have begun to partner with external organisations to alleviate the administrative burden on health personnel and to ensure that information is collected and analysed efficiently [3, 29, 32–34]. External organisations may include local academic institutions or neighbouring health departments who have the time, skills and necessary knowledge to accurately document and assess a disaster response. The analytical skills of the external partners acts as the mechanism by which to produce thorough documentation and objective assessments of a health department’s response to a disaster [26, 33].

### Demi-regularities identified in the ‘disseminate’ stage of the monitoring and evaluation cycle

There was one demi-regularity identified within the disseminate stage of the M&E cycle. This demi-regularity can be summarised as follows, ‘The standardisation of M&E reports is likely to promote inter-agency learning’. The broad dissemination of evaluation reports is critical to building a body of evidence from which all health departments can learn. However, there is currently a high degree of variability in the structure and terminology used in evaluation reports, alongside gaps in the quality and completeness of data [13, 35, 36]. Such inconsistencies make it difficult for one health department to perceive the experiences of another as relevant to their own work [4, 13]. The dissemination of standardised reports would allow all health departments to acknowledge how the lessons learned by another department may be applicable to their own. This mechanism would: support comparisons across settings and over time; promote inter-agency learning; and help health departments to continually refine their response procedures [23, 26].

### Demi-regularities identified in the ‘implement’ stage of the monitoring and evaluation cycle

There were two demi-regularities identified within the implement stage of the M&E cycle.

The first demi-regularity in the implement stage was, ‘Positive leadership attributes at the management level increases the likelihood that changes to improve response procedures will be successfully implemented’. The evidence synthesised in this RRR clearly demonstrates that well-defined leadership is critical to the success of implementation activities. We found substantial evidence from the literature which supported the notion that leaders who foster cooperation, value input from staff, empower employees, are enthusiastic and provide constructive feedback are crucial to facilitating an organisational culture of quality improvement [22, 37, 38]. We postulate that the establishment of a culture of quality improvement is a mechanism that can increase motivation to refine disaster response processes.

The second demi-regularity in the implement stage was, ‘Shared decision making structures are likely to promote successful implementation processes’. Creating sustainable change in an emergency response organisation can be a highly complex and difficult task [13, 22]. We found that decision making structures play a significant role in influencing how health personnel respond to activities and also to organisational changes. Involving health personnel when allocating roles and responsibilities in disaster scenarios facilitates a sense of equity, ensures buy-in and also encourages a sense of responsibility [28]. Furthermore, involving health personnel in planning implementation activities ensures that specific needs and resources are considered and that the proposed changes are achievable [3, 13, 34]. This context of shared decision making triggers a mechanism that fosters a sense of ownership amongst health personnel. This, in turn, facilitates the successful adoption and execution of implementation activities by health personnel.

## Discussion

### Summary of findings

Local health departments are often at the forefront of a disaster response delivering lifesaving health services. M&E can be used to help health departments reflect on their response efforts to learn from what worked and what did not and to identify ways to improve their response to future events. However, the literature indicates that many health departments struggle to effectively learn from past mistakes, thus the same errors are often repeated time and again [4, 12–15]. We conducted this RRR to understand how health departments can better use M&E to successfully learn from past experiences and improve disaster response procedures.

In the first stage of our M&E cycle, document and assess, we uncovered several CMO configurations which were likely to encourage thorough documentation and assessment of the disaster response. Firstly, there was strong support in the literature for health departments to assure staff that they would not be penalised for mistakes. This was found to create a culture of learning whereby staff felt comfortable to admit to mistakes and report response errors. This mechanism would lead to more accurate documentation of the response. Secondly, embedded training methods provide health personnel with regular opportunities to practice documenting and assessing their work. This leads to staff being more familiar with documentation procedures and is likely to translate into higher quality documentation and assessment of a disaster response. Alternatively, using external partnerships is likely to improve the depth and quality of reports as such partners have the necessary skills to document and assess a response.

Dissemination is a crucial component of M&E which promotes the sharing of lessons to a broad range of stakeholders. However, the literature indicated that dissemination was one of the weakest points in the application of M&E in the disaster context. Despite this recurrent finding, there appeared to be very limited research into why this was the case and how dissemination could be improved. Due to this gap in the literature, we were only able to analyse one demi-regularity. This mechanism works by allowing personnel from one department to understand and perceive the experiences of others as relevant to their own. Such a mechanism would be triggered when the disseminated reports are standardised in both format and terminology, thereby promoting inter-agency learning. It is acknowledged that this mechanism is heavily influenced by factors external to a health department. For example, there would need to be a governing body who oversees the dissemination of all reports to ensure they are written to standard. This would require a sustained commitment and administrative effort which not all governments will have the capacity for. Further research into how health departments in LMICs can broadly disseminate their lessons is needed. At the implementation stage of the M&E cycle, positive leadership attributes and shared decision making structures were both found to promote successful outcomes by motivating staff and also fostering a sense of responsibility and ownership among health personnel.

This RRR revealed that there are very few studies which have investigated experiences with M&E in LMICs, despite these countries being disproportionately affected by disasters. This is consistent with the more general findings of Gocotano and colleagues (2015) who found that there are fewer papers documenting disaster or emergency events in low-income countries compared to high-income countries [39]. Only 2 of the 30 papers included in this RRR analysed the experiences of health departments in LMICs [20, 38], while the remaining literature focused on high-income countries (the United States in particular). It has recently been noted that even when papers are published on events in low-income countries, they are often written by authors with institutional affiliations external to the country of the event [39]. These factors may mean that there are CMO configurations unique to the experience of local personnel working in health departments in low resource countries which are not adequately reflected in the literature we have analysed. Although many of the CMO configurations synthesised in this RRR have been drawn from literature on disaster and emergency health events in high-income countries, the findings are applicable and transferrable to other setting due to the ‘all-hazard approach’ in disaster management, which emphasises that important systems issues will be common across disasters [21].

### Strengths and limitations

A strength of this RRR lies in its utilisation of realist methodology, in particular the use of the CMO configurations, to understanding not only whether an intervention works or not but also how and why an intervention (i.e., M&E) may work. Other evidence synthesis approaches used for informing policy decisions, such as meta-analyses or narrative studies, have key limitations which prevent an explanation of how an intervention works and how it may operate in a different setting [16]. This RRR used a heuristic approach to examine the existing literature describing health departments’ experiences with M&E. A synthesis of this evidence identified a number of mechanisms that may facilitate or hinder the M&E process. The identification of these mechanisms is in itself of great value to health departments implementing M&E. However further value is gained from this RRR through the theory driven and explanatory nature of realist evaluation. This approach enabled the construction of CMO configurations that explain exactly how and why M&E can be successfully used to learn lessons and improve future disaster response efforts. Although available evidence lacked sufficient detail for us to conduct a more comprehensive examination of what works and does not work at each stage of the disaster recovery cycle, we were able to draw lessons that can be applied across the board.

A possible limitation of this review is that some of the CMO configurations identified are, at this stage, based on the recommendations or ideas of the original authors’ of the reviewed papers, rather than tested experiences of M&E. This may reflect either a shortage of health departments engaging with M&E to learn lessons from disaster response efforts or a gap in research investigating health departments’ experiences with M&E. Nevertheless, the inferences drawn in this RRR are clearly acknowledged and based on a rigorous analysis of the literature currently available. We have aimed for transparency by documenting which papers contributed to our synthesis of CMO configurations (Table 4) and advise readers to carefully consider how these CMO configurations relate to their situation.

**Table 4:** Papers which contributed to final analysis

Stage of theoretical framework	Document reference	Country setting	Type of disaster	Level of health system
Document and Assess				
	Green et al. 2003 [20]	Guatemala	Disaster scenario - propane tank explosion at an open-air food vendor	Hospital
	Klein et al. 2005 [34]	USA	Bioterrorism	Hospital
	Macrae 2014	UK	Healthcare disasters	Hospital
	Seale 2010 [28]	USA	Hurricanes (Rita 2005, Ike 2008)	Post-acute rehabilitation facility
	Biddinger et al. 2008 [32]	USA	All public health emergencies	State and local public health departments
	Nelson, Lurie & Wasserman 2007 [23]	USA	All disasters	Public health system
	Piltch-Loeb 2014a [26]	USA	All disasters	Public health system
	Piltch-Loeb 2014b [33]	USA	All disasters	Public health system
	Savoia, Agboola & Biddinger 2012 [14]	USA	H1N1 pandemic and hurricanes (Ike 2008, Gustav 2008, Katrina 2005)	Public health system
	Seid et al. 2007 [22]	USA	All disasters	Public health system
	Stebbins & Vukotich 2010 [29]	USA	Hepatitis A outbreak	Public health department
	Adini et al. 2013 [3]	Israel	Mass casualty incidents	National health system
	^a^Birkland 2009 [30]	USA	All disasters	Not health specific
	^a^Donohue & Tuohy 2006 [13]	USA	All disasters	Not health specific
	^a^Dufty 2013 [31]	Australia	All emergencies	Not health specific
	^a^Spillsbury et al. 2007 [27]	Not country specific	Not disaster specific	Not health specific
Disseminate				
	Mears et al. 2010 [35]	USA	All public health emergencies	State public health system
	Nelson, Lurie & Wasserman 2007 [22]	USA	All disasters	Public health system
	Piltch-Loeb 2014a [26]	USA	All disasters	Public health system
	Savoia, Preston & Biddinger 2013 [36]	USA	All public health emergencies	Public health system
	^a^Donohue & Tuohy 2006 [13]	USA	All disasters	Not health specific
Implement				
	Adini et al. 2012 [21]	Israel	Mass casualty events	Hospital
	Klein et al. 2005 [34]	USA	Bioterrorism	Hospital
	Bevc et al. 2012 [37]	USA	All disasters	Local health department
	Chan et al. 2010 [38]	Indonesia	Tsunami	Local health clinics
	Seale 2010	USA	Hurricanes (Rita 2005, Ike 2008)	Post-acute rehabilitation facility
	Seid et al. 2007 [22]	USA	All disasters	Public health system
	Adini et al. 2013 [3]	Israel	Mass casualty incidents	National health system
	^a^Batalden & Davidoff 2007	Not country specific	Not disaster specific	Healthcare systems
	^a^Donohue & Tuohy 2006 [13]	USA	All disasters	Not health specific

^a^Indicates papers that were provided by the local reference group

During the search process, we excluded non-health research to ensure that we would only evaluate issues relevant to health departments when conducting M&E. However, several papers provided by the local reference group focussed on other sectors, particularly high-risk industries such as aviation, which have made significant progress with quality improvement approaches such as M&E [13, 22, 30, 33]. We suggest that future research consider how other sectors have been able to refine their M&E practices. Investigation outside of the health sector could reveal transferable theories that may assist local health departments to improve M&E practices.

## Conclusion

Despite the sparse literature, it was evident that local health departments who engage with M&E processes to evaluate their responses to major disasters often fail to improve future response efforts. This paper summarises the CMO configurations that were found to have an effect on outcomes at each stage of the M&E cycle (document and assess, disseminate and implement). We have considered how these may act to constrain or enable the ability of people using M&E approaches to effectively learn lessons from previous experiences. Firstly, to support thorough and accurate documentation and assessment of a disaster response, local health departments should consider how they can: create a culture of learning within health departments; use embedded training methods; or facilitate external partnerships. Secondly, to enhance the widespread dissemination of lessons learned from a disaster response effort, evaluation reports should be standardised in format and terminology. Lastly, to increase commitment to the implementation of improvement processes, local health department leaders should display positive leadership attributes and encourage shared decision making.

Further research relevant to disaster prone regions is critical to enabling additional CMO configurations to be uncovered and to contribute to the understanding of how local health departments can use M&E to effectively learn from the past. For example, there was some emerging evidence [13, 26, 29] which suggested that digital platforms could be used to assist in the broad dissemination of lessons learned from disaster response efforts. Further exploration of this idea was hindered by the paucity of evidence in the literature. Future research should analyse how new technologies may be utilised in M&E to improve disaster response efforts.

This study is among the first to conduct a synthesis of the CMO configurations which facilitate or hinder M&E efforts aimed at improving future disaster responses. It makes a significant contribution to the disaster literature and provides an evidence base to help health departments to understand how they may successfully use M&E to improve disaster response efforts. Our findings and recommendations will be particularly useful for health departments in LMICs of East Asia and the Pacific who are frequently affected by disasters and stand to gain the most from rigorous and purposeful disaster M&E.

## Abbreviations

CMO: Context-mechanism-outcome
LMIC: Low and middle income country
M&E: Monitoring and evaluation
RRR: Rapid realist review
